# From multitude to singularity: An up-to-date overview of scRNA-seq data generation and analysis

**DOI:** 10.3389/fgene.2022.994069

**Published:** 2022-10-03

**Authors:** Giulia Carangelo, Alberto Magi, Roberto Semeraro

**Affiliations:** ^1^ Department of Experimental and Clinical Biomedical Sciences “Mario Serio”, University of Florence, Florence, Italy; ^2^ Department of Information Engineering, University of Florence, Florence, Italy; ^3^ Department of Experimental and Clinical Medicine, University of Florence, Florence, Italy

**Keywords:** single cell, RNA sequencing, transcriptomics, spatial transcriptomics, biomedical applications, technological evolution

## Abstract

Single cell RNA sequencing (scRNA-seq) is today a common and powerful technology in biomedical research settings, allowing to profile the whole transcriptome of a very large number of individual cells and reveal the heterogeneity of complex clinical samples. Traditionally, cells have been classified by their morphology or by expression of certain proteins in functionally distinct settings. The advent of next generation sequencing (NGS) technologies paved the way for the detection and quantitative analysis of cellular content. In this context, transcriptome quantification techniques made their advent, starting from the bulk RNA sequencing, unable to dissect the heterogeneity of a sample, and moving to the first single cell techniques capable of analyzing a small number of cells (1–100), arriving at the current single cell techniques able to generate hundreds of thousands of cells. As experimental protocols have improved rapidly, computational workflows for processing the data have also been refined, opening up to novel methods capable of scaling computational times more favorably with the dataset size and making scRNA-seq much better suited for biomedical research. In this perspective, we will highlight the key technological and computational developments which have enabled the analysis of this growing data, making the scRNA-seq a handy tool in clinical applications.

## 1 Introduction

For many years researchers have tried to comprehend the complexity of tissues, organs and organisms (Grizzi and Chiriva-Internati, 2005). In order to gain this understanding, many studies have focused on cell characterization, redefining the cell as not only the structural but also the functional unit of life (Arendt et al., 2016).

Traditionally, cells have been classified by their morphology or by the expression of certain proteins in functionally distinct settings, but the advent of NGS techniques paved the way for the detection and quantitative analysis of cellular content (Mosmann et al., 1986; Orkin, 2000; Poulin et al., 2016). The high amount of data generated in modern genomics and transcriptomics experiments permitted to better characterize the architecture of genomes and the complexity of the molecular mechanisms underlying cellular activity, allowing an increasingly more accurate and in-depth depiction of cell plasticity in dynamic processes such as development, differentiation and disease evolution (Sedlazeck et al., 2018; Stark et al., 2019).

Modern cellular and molecular biology knowledge is largely derived from RNA sequencing (RNA-seq) experiments. Over the last 20 years, the transcriptome quantification has shaped our understanding of mechanisms responsible for phenomena, such as the alternativeness of the mRNA splicing process, the regulation of gene expression by non-coding and enhancer RNAs respectively and the drug resistance in some types of cancer, becoming a common and powerful technology suitable for biomedical research (Wang et al., 2008; Morris and Mattick, 2014; Li et al., 2016; Marco-Puche et al., 2019).

The adaptation and evolution of RNA-seq has been driven by technological developments and resulted in a progressive increase of the analysis resolution. Starting from the so called “bulk” RNA-seq, capable of measuring the average gene expression levels of ensembles of millions of cells, we moved to the scRNA-seq that, by allowing to profile the transcriptome of single cells, has revealed rare cellular properties and biologically meaningful cell-to-cell variability, laying the groundwork for heterogeneity-oriented studies (Svensson et al., 2018; Li and Wang, 2021).

As experimental protocols have improved rapidly, computational workflows for processing the data have also been refined, taking into account the increased throughput of scRNA-seq experiments (Andrews et al., 2021). The current “standard” analysis pipeline consists of two main sections: preprocessing, including all the steps necessary to clean the data matrix from unwanted sources of information (quality control, normalization, data correction, feature selection and dimensionality reduction) and cell- and gene-level downstream analysis, used to extract biological insights and describe the underlying biological system. For each of these steps, computational biologists developed a range of methods which perform better in different tasks and settings, making the creation of generalizable workflows for single cell experiments analysis challenging.

In this perspective, we will present an overview of the computational workflow, arguing the tools available to proceed in each step and highlighting the key technological developments which have enabled the analysis of this ever-increasing amount of data, making the scRNA-seq a handy tool in biomedical research.

## 2 Single cell sequencing

**FIGURE 1:**
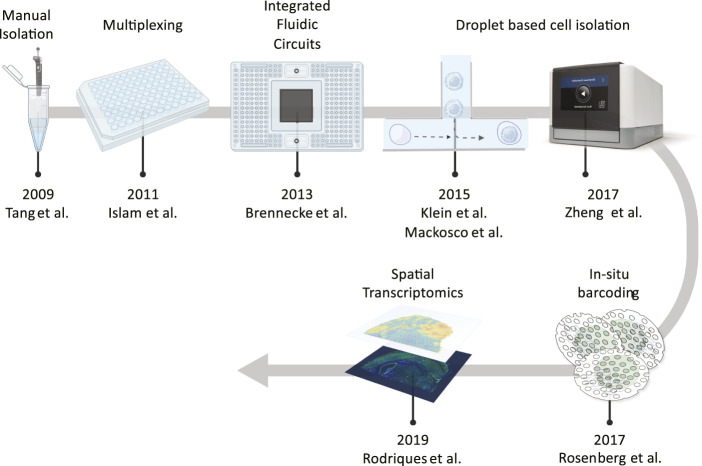
Noteworthy technologies that have allowed to profile large numbers of cells in parallel. Starting from manual isolation methods, a jump to ∼100 cells was enabled by sample multiplexing and than the development of integrated fluidic circuits increased these numbers to an order of magnitude. Next, the introduction of nanodroplet technologies increased throughput even further to hundreds of thousands of cells, as for *in situ* barcoding which favoured the development of spatial methods.

The first studies of single cells date back to the early 90s and were motivated by incoming discoveries which highlighted cell plasticity in dynamic processes and the different functionality based on localization (Eberwine et al., 1992). The advent of NGS techniques opened up to the era of quantitative analysis of cellular content, although first transcriptomics techniques (bulk RNA-seq) were not able to survey the diversity of cell types in a sample (Hong et al., 2020). The scaling of technologies to profile large numbers of cells in parallel has been the key to driving single cell transcriptomics forward (see Figure 1).

### 2.1 Technical evolution

The first example of single cell transcriptomics is the study of a handful of mouse primordial germ cells by Tang et al. (2009). By manual modification of cDNA amplification protocols previously employed in microarray analyses, he captured and quantified for the first time the full-length cDNAs for 64% of the expressed genes of a single cell, without affecting the accuracy of the protocol, which was however very time consuming and limited to small numbers of atypically large cells.

In the wake of Tang et al., new different approaches were developed including the so-called tag sequencing methods. For instance, in 2011, Islam et al. quantified the transcriptome of 85 cells by means of single cell tagged reverse transcription (STRT) (Islam et al., 2011). In brief, the authors settled single cells into the wells of a 96-well PCR plate preloaded with lysis buffer and then added reverse transcription (RT) reagents to generate a first-strand cDNA. Next, a unique template-switching oligo (TSO) with a specific sequence (six-base) on its 3′ end and a universal primer sequence on the 5′ was added to each well triggering the RT template-switching mechanism which produces a cDNA molecule incorporating the sequence at the 3′ of the TSO.

The introduction of these “barcode” sequences allowed, for the first time, to assay many cells in parallel *via* multiplexed unbiased RNA-seq, although, in the STRT-seq method, full-length cDNA is amplified by template-switching, but only the 5′ end fragment is captured and sequenced. To overcome thislimitation, the full-length SMART-seq (Ramsköld et al., 2012) and SMART-seq2 (Picelli et al., 2013) protocols were developed by Ramsköld et al. and Picelli et al., in 2011 and 2013 respectively. Compared with existing tag based methods, SMART-seq has improved read coverage across transcripts, promoting a detailed analyses of alternative transcript isoforms and identification of single-nucleotide polymorphisms (SNP).

In sight of this, it is therefore necessary to clarify that it is possible to profile the transcriptome through full-length transcript analysis or by digital counting of either 3′ or 5′ ends. While the two methods carry similar levels of reproducibility, the latter methods consist in a cost-effective solution to quantify a high amount of transcripts at the expense of a large loss of information for each of these, contrary to the former which, by taking advantage of full-length transcripts entirety, allows the detection of splice variants and alternative transcripts, as well as genetic alterations in the transcribed fraction but for a lower number of cells.

A further application of SMART-seq2 protocols, although with some modifications (Egidio et al., 2014), is found also in the work of Brennecke et al. (2013). By means of an integrated fluidic circuit (IFC) method, implemented in the Fluidigm C1 system, they studied 96 cells isolated into individual reaction chambers and subjected to automatic staining, lysis, and sequencing in extraordinarily fast times and in a “passive” manner never seen before. In fact, the key feature of this technology is the design of microfluidics devices (or chips) that allow the sequential delivery of very small and precise volumes into tiny reaction chambers. However, a major limitation derives from the number of these chambers (96) which restrict the analysis to an equivalent number of cells, as for Brennecke in 2013. Some following large-scale studies made use of a large number of IFCs to create big data sets (Zeisel et al., 2015).

In 2015, the advent of microfluidic platforms bypassed this drawback thanks to the usage of nanoliter microreactor droplets which can encapsulate cells with no physical, and therefore numerical, restraints. The inDrop (Klein et al., 2015) and the Drop-seq (Macosko et al., 2015) protocols enter the scene with related commercial systems that allow to randomly capture cells in beads containing lysis buffer, RT reagents and barcoded oligonucleotide primers, so that mRNA is released from each cell and remains trapped in the bead to be barcoded during synthesis of cDNA. The two methods mainly differ in barcoding strategy and amplification technique, since the inDrop protocol uses hydrogel beads bearing poly(T) primers with defined barcodes and, after pooling, initiates linear amplification (IVT), contrary to Drop-seq which uses beads with random barcodes and amplifies through PCR. The random isolation of cells, however, comes with inherent limitations. Poisson statistics of cell capture to ensure that mostly single cells are isolated means there will always be large inefficiencies in terms of cell isolation, and the pool of barcodes will always have to be substantially larger than the number of cells captured to avoid barcode duplication. A large number of barcodes means the usage of very long and therefore expensive oligos. To reduce their synthesis costs, two different strategies are adopted by both methods: the combination of multiple shorter designed barcodes (e.g., 8–10 bases) into longer barcodes (e.g., 8 bases +22-base linker +10 bases = 40 bases), as for InDrop, or the synthesis of very long (e.g., 12 bases) random barcodes, as for DropSeq. This second procedure is simpler than the first and does not require any synthesized oligos for the barcodes. However, in the first approach barcodes can be designed to avoid biases and ensure that each sequence will be distinct.

The need for a large number of oligos was mitigated in 2017, through the advent of the combinatorial *in situ* barcoding methods, when Rosenberg et al. introduced the split-pool ligation-based transcriptome sequencing (SPLiT-seq), a low-cost, scRNA-seq method that enables transcriptional profiling of hundreds of thousands of fixed cells or nuclei in a single experiment (Rosenberg et al., 2018). In brief, a suspension of formaldehyde-fixed cells or nuclei passes through four rounds of combinatorial barcoding. At the first round, cells are distributed in a 96-wells plate and labelled with a specific tag. Next, cells are pooled and subjected to another label-expanding round. So, in the third round, another portion is added, carrying with it a unique molecular identifier (UMI) specific for each transcript and also used in other tag-based methods, such as STRT-seq, InDrop and Drop-seq, to better quantify the native, unamplified transcript levels (Islam et al., 2014; Stegle et al., 2015). Finally, sequencing adapters are introduced by PCR and, subsequently, each transcriptome is assembled by combining reads containing the same four-barcode combination.

Along with SPLiT-seq, one of the most vastly used methods makes its entry. The 10x Genomics company presents a new system called Chromium, based on an inDrop-seq variant. Specifically, single cells, RT reagents, Gel Beads containing barcoded oligonucleotides, and oil are combined onto a microfluidic chip to form reaction vesicles called Gel Beads in Emulsion, or GEMs. GEMs are formed in parallel within the 8 microfluidic channels of the chip, allowing the user to process hundreds to hundreds of thousands of single cells in a single 7-min run, with a ∼65% of capture efficiency (Zheng et al., 2017). Within each GEM reaction vesicle, a single cell is lysed, the Gel Bead is dissolved to free the identically barcoded RT oligonucleotides into solution, and reverse transcription of polyadenylated mRNA occurs. As a result, all cDNAs from a single cell will have the same barcode, allowing the sequencing reads to be mapped back to their single cells of origin. The scalability and robustness of the system has favored the rapid diffusion of this device and its acquisition by many research laboratories in the medical field. Another contribution to this field comes from the so-called spatial RNA sequencing (spRNA-seq). Introduced in 2019 to enable the understanding of how tumor cells can communicate with each other, escape the immune system, develop drug resistance and metastasize, it combines the strengths of the global transcriptional analysis of bulk RNA-seq and *in situ* hybridization, providing whole transcriptome data with spatial information. Two technologies are currently available by 10x Genomics and Nanostring Technologies, both using proprietary spatial gene expression slides on which to fix fresh-frozen or Formalin-Fixed Paraffin-Embedded (FFPE) tissue. The two technologies differ for slide functionalization. The 10x device contains oligo capture probes, similar to those coating the gel beads, and once the tissue is fixed, stained and imaged, it is permeabilized to release the RNA, captured by probes and subjected to on-slide cDNA synthesis (Ståhl et al., 2016; Rodriques et al., 2019). The Nanostring system, uses barcode-labeled probes and fluorescent markers to hybridize to mRNA targets and to establish tissue “geography” respectively. After the regions-of-interest (ROIs) are selected, the barcodes are released *via* UV exposure and collected from the ROIs on the tissue (Moses and Pachter, 2022).

The labeled RNAs, for both technologies, are then sequenced through standard NGS procedures.

The spRNA-seq is still in its early stages and there are several common challenges that limit its applications, including non-single cell resolution, relatively low sensitivity, high cost and labor-intensive process, but given its capacity to dissect intercellular subpopulations sensitively and spatially, it will inevitably become a fundamental area of research in both discovery and therapeutics.

### 2.2 Bioinformatic analysis

#### 2.2.1 General information and workflow

The rapid technological evolution that allowed the parallel analysis of thousands of cells, promoting the spread of scRNA-seq techniques, was accompanied by the development of new data analysis pipelines capable of managing such a large amount of data. The mathematical representation of these massive datasets is an “expression” matrix, defined by the number of detected genes and observed cells respectively. The process aimed at its generation starts with the read quality check. The FastQ files outputted from the sequencer are evaluated by means of quality check tools, like FastQC (https://www.bioinformatics.babraham.ac.uk/projects/fastqc/), to undergo de-multiplexing, adapter trimming, alignment and count. Tailored pipelines such as Cell Ranger (Zheng et al., 2017), UMI-tools (Smith et al., 2017), scPipe (Tian et al., 2018) and zUMIs (Parekh et al., 2018), were developed to carry out these preliminary steps. Alternatively, researchers can build their own workflows by combining individual methods that address each of the aforementioned tasks (see Table 1). For instance, the STAR (Dobin et al., 2013) aligner implements the STARsolo algorithm suited to trim, align and count this kind of data in a very fast way (Brüning et al., 2022).

**TABLE 1:** Raw data processing tools.

	Name	Alignment	QC	Count	CC	PL	References
Pipelines	CellRanger	x	x	x	x	R/Python	Zheng et al. (2017)
	UMI-tools	x	x	x	x	Python	Smith et al. (2017)
	scPipe	x	x	x	x	C++/R	Tian et al. (2018)
	zUMIs	x	x	x	x	R/Perl	Parekh et al. (2018)
	dropEst	x	x	x	x	C++	Petukhov et al. (2018)
	Optimus	x	x	x	x	Python/C++	
Tools	STAR	x	x	x	x	C/C++	Dobin et al. (2013)
	HISAT2	x	-	-	-	C/C++	Kim et al. (2015)
	kallisto	-	-	x	-	C/C++	Bray et al. (2016)
	FastQC	-	x	-	-	Java
	HTSeq	-	x	x	-	Python	Putri et al. (2022)
	featureCount	-	-	x	-	C	Liao et al. (2014)
	EmptyDrops	-	-	-	x	R	Lun et al. (2019)

QC, quality check; CC, cell calling; PL, programming language.

**FIGURE 2:**
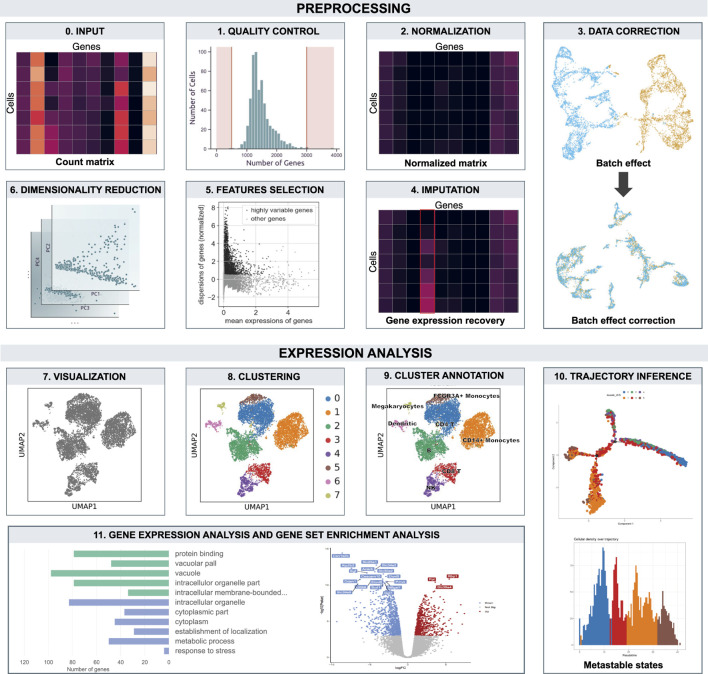
Overview of the workflow. The count matrix undergo preprocessing and expression analysis. Boxes are ordered according data analysis flow.

Moreover, if reads are UMI-tagged, only cell barcodes that represent intact individual cells are kept. The most unambiguous approach to assess emptiness is to calculate a dataset-specific threshold of the minimum number of UMIs required to consider a barcode as a cell (Zheng et al., 2017). Alternatively tools, such as EmptyDrops (Lun et al., 2016a), identify cell barcodes that significantly deviate from background levels of RNA present in empty wells. The resulting cells still show unwanted biases. All processes involved in bias removal define the so called “preprocessing” which consists in quality control, normalization, batch correction, feature selection and dimensionality reduction. All these steps are preparatory for the following expression analysis, used to extract biological insights and describe the underlying biological system (see Figure 2).

Also in this context, tailored pipelines and individual tools are available to perform each operation. Toolboxes, such as Scanpy (Wolf et al., 2018), SCell (Diaz et al., 2016), Seurat (Hao et al., 2021) and scater (McCarthy et al., 2017) allow to complete multiple tasks bypassing problems related to data format conversions, making the analysis simpler. On the other hand, it is important to remember that it is difficult for a tool with many functions to continue to represent the state of the art in all of them.

In this perspective, we will present an overview of the computational workflow, arguing the tools available to proceed in each step (see Table 2).

**TABLE 2:** Analysis tools.

		Preprocessing				Expression analysis				PL	
	Name	QC	N	BC	DR	V	C	DE	TI		References
Pipelines	CellRanger	x	x	x	x	x	x	x	-	R/Python	Zheng et al. (2017)	
	Scanpy	x	x	x	x	x	x	x	x	Python	Wolf et al. (2018)	
	Seurat	x	x	x	x	x	x	x	-	R	Hao et al. (2021)	
	SCell	x	x	x	x	x	x	x	x	Matlab	Diaz et al. (2016)	
	scater	x	x	x	x	x	x	x	x	R	McCarthy et al. (2017)	
	Pagoda2	x	x	x	x	x	x	x	-	R	Lopez et al. (2018)	
Tools	Doublet Finder	x	-	-	-	-	-	-	-	R	McGinnis et al. (2019)	
	Scrublet	x	-	-	-	-	-	-	-	Python	Wolock et al. (2019)	
	scds	x	-	-	-	-	-	-	-	R	Bais and Kostka (2020)	
	scran	x	x	-	-	-	-	-	-	R	Bray et al. (2016)	
	SCnorm	-	x	-	-	-	-	-	-	R
	bioinfokit	-	x	-	-	-	-	-	-	R	Putri et al. (2022)	
	ComBat	-	-	x	-	-	-	-	-	R	Johnson et al. (2007)	
	mnnCorrect	-	-	x	-	-	-	-	-	R	Haghverdi et al. (2018)	
	Harmony	-	-	x	-	-	-	-	-	R	Korsunsky et al. (2019)	
	BBKNN	-	-	x	-	-	-	-	-	Python	Polański et al. (2020)	
	SAUCIE	-	-	x	x	x	x	-	-	Python	Amodio et al. (2019)	
	scVI	-	-	x	x	-	-	x	-	Python	Boyeau et al. (2019)	
	PCA	-	-	-	x	-	-	-	-	Python	Pedregosa et al. (2011)	
	t-SNE	-	-	-	x	x	-	-	-	Python/R	Van der Maaten and Hinton (2008)	
	UMAP	-	-	-	x	x	-	-	-	Python/R	McInnes et al. (2018)	
	Louvain	-	-	-	-	-	x	-	-	Python/R	Blondel et al. (2008)	
	Leiden	-	-	-	-	-	x	-	-	Python/R	Traag et al. (2019)	
	MAST	-	-	-	-	-	-	x	-	R	Finak et al. (2015)	
	scCODE	-	-	-	-	-	-	x	-	R	Zou et al. (2022)	
	Slingshot	-	-	-	-	-	-	-	x	R	Street et al. (2018)	
	DPT	-	-	-	-	-	-	-	x	Python	Haghverdi et al. (2016)	
	Whishbone	-	x	-	x	x	-	x	x	Python	Setty et al. (2016)	
	Monocle2	-	x	x	x	x	x	x	x	R	Trapnell et al. (2014)	
	Monocle3	-	x	x	x	x	x	x	x	R	Cao et al. (2019)	
	velocyto	-	x	x	x	x	x	x	x	Python/R	La Manno et al. (2018)	
	scVelo	-	x	x	x	x	x	x	x	Python	Bergen et al. (2020)	

QC, quality check; N, normalization; BC, batch correction; DR, dimensionality reduction; V, visualization; C, clustering; DE, differential expression; TI, trajectory inference; PL, programming language.

##### 2.2.1.1 Quality control

Before analyzing the expression matrix, we must assess the uniqueness of each barcode and cell viability. To this end, it is important to keep in mind that some droplets might contain more than 1 cell or no cell at all, making it a doublet, multiplet or an empty droplet. Furthermore, cells can be dying or damaged during isolation, misrepresenting the sample composition. So, we need to filter out them.

A possible solution is to identify these cells by evaluating three aspects of the data: the number of counts per cell/barcode (count depth), the number of genes per cell/barcode, and the fraction of counts from mitochondrial genes per cell/barcode. The thresholds for these covariates are arbitrary based on the general characteristics of the data itself, but they allow us to filter out cells with low count depths, few detected genes and/or high fraction of mitochondrial counts, as those are considered damaged cells, and at the same time they allow to filter out cells with too high counts which are indicative of doublets or multiplets (Ilicic et al., 2016).

However, a misinterpretation of these covariates could lead to wrong filtering, since in some cases a deviation in one of these values may be related to a particular cell condition, such as heavy respiration (high mitochondrial counts), quiescence (low counts, few genes) and a larger size (high counts). Therefore, they should be considered jointly when univariate thresholding decisions are made, and these thresholds should be set as permissive as possible to avoid filtering out viable cell populations unintentionally.

For doublet detection, more precise methods were developed (Xiong et al., 2022). For instance, scrublet (Wolock et al., 2019) is able to discern “embedded” (same cell type) from “neotypic” (different cell types) doublets, assuming that among all observed transcriptomes, multiplets are relatively rare events and that all cell states contributing to doublets are also present as single cells elsewhere in the data.

Quality control can also include a gene filtering step, since genes expressed in few cells are non-informative of the cellular heterogeneity. The threshold is again arbitrary, but in principle it should scale with the number of cells in the dataset and the intended downstream analysis, because, based on that choice, for example, it could limiti the identification of small clusters that might actually carry valuable information about less represented cell population.

##### 2.2.1.2 Normalization

By means of quality control we removed sources of unwanted and inaccurate information. However, the dataset is still affected by multiple biases due to technical and biological variability. Sources responsible for such events could be, for example, capture efficiency, amplification and incomplete library sequencing. The consequence is an alteration in the counts which make cells incomparable (Macosko et al., 2015).

Normalization addresses this issue by e.g., scaling count data to obtain correct relative gene expression abundances between cells. Available methods can be linear or non-linear: a linear approach involves the estimation of size factors based on a linear regression over genes, while non-linear methods usually apply parametric modelling on count data and correlate technical and biological sources of variability to correct both (Lytal et al., 2020).

The most common normalization approach is the count depth scaling by “counts per million” (CPM), which operates by dividing gene counts by the total number of mapped reads per sample and multiplying by 1 × 10^6^. CPM falls within linear global scaling normalization methods and assumes that all cells in the dataset initially contained an equal number of mRNA molecules (10^6^) and count depth differences arise only due to sampling. Variations of this method scale the size factors with different factors of 10, or by the median count depth per cell in the dataset. Tools such as scran (Lun et al., 2016b) and Scanpy implement extensions of CPM approach. The former was proven to perform better than others in order to proceed with differential expression (DE) analysis (Vieth et al., 2019).

For datasets with strong batch effects, non-linear methods were proven to be more reliable, particularly for plate-based scRNA-seq data, usually affected by batch effect between plates (Svensson et al., 2017).

For full-length sequencing protocols, methods which consider the gene length are more suitable. The most common is “transcripts per million” method (TPM), implemented, for example, in the bioinfokit toolbox (http://doi.org/10.5281/zenodo.3698145) (Putri et al., 2022).

Another crucial factor for normalization is the presence of synthetic spike-ins or UMIs as a means to correct for amplification bias. By adding known concentrations of external transcripts, called spike-ins, it is possible to evaluate the presence of technical artifacts, looking for differences between their observed and expected expression. By calculating a cell-specific factor that adjusts for the differences, and by applying that factor to endogenous genes, normalized expression estimates can be obtained. In spite of the promise, there are many challenges in getting spike-ins to work well, which can result in inconsistent detections (Grün et al., 2014). Contrary to spike-ins, UMIs are easier to handle since they are attached to individual transcripts prior to PCR, making each molecule unique and allowing an absolute molecular count (Kivioja et al., 2011).

Also, genes can be normalized to make them comparable between cells. Gene counts can be scaled to have a zero mean and a unit variance (z-score), making genes equally weighted. The scaling is currently not a routine because sometimes it could be useful to give genes the same weight and sometimes not, due to the effect produced by an expression magnitude difference.

Normalized data should be log (x+1)-transformed for use with following analysis methods that assume data are normally distributed. Three main effects derive from this transformation: log values represent log fold changes (unit to measure expression), they become normally distributed, reducing the skewness of the data and finally, the mean-variance relationship typical of single cell data is mitigated (Brennecke et al., 2013).

##### 2.2.1.3 Batch Correction

Through the normalization, we mitigated the sources of technical variability responsible for gene counts alterations. However, the dataset may still contains unwanted signals of technical and biological nature. In the latter category falls for e.g., the cell cycle effect, while in the former, the batch effect deriving from different experimental protocols or/and different plates.

In order to get rid of these biases, it is possible to proceed with data and batch correction. Currently, several tools can accomplish these tasks with different approaches (Chu et al., 2022). For example, in development-oriented studies regressing out the cell cycle effect could uncover the desired biological signals (Vento-Tormo et al., 2018; Büttner et al., 2019). To this end, methods such as Scanpy and Seurat implement functions to score the cell cycle phases and regress linearly their biological effect. Alternatively, tailored tools based on complex models, like f-scLVM (Buettner et al., 2017), are available. Sometimes, also the count bias produced by differences in cell size, if not enough corrected through normalization, could be further mitigated to emphasize development-related signals. In this situation, regressing both covariates at the same time could be the best solution to account for dependence between them.

Correcting for biological biases, however, it is not always necessary or useful, since they can be avoided through pondered experimental design or because they can relate to the biological process of interest. The same observation is in part valid also for those of technical nature. In fact, even in this case a clever experimental design allows to reduce their influence but, if present, they have no correlation with the biological signals, so they must be mitigated. This process, named batch correction, can be conducted between samples and cells of the same experiment through linear models, or among different datasets derived from multiple experimental settings through non-linear models.

One of the most common linear methods is ComBat (Johnson et al., 2007) which take into account the batch effect on mean and variance of the dataset, performing very well in most settings (Büttner et al., 2019).

If the differences in the datasets are more pronounced, linear models could confound the intra- and inter-technical and biological biases, and in this circumstances non-linear models implemented in tools such as Canonical Correlation Analysis (CCA) (Butler et al., 2018), Mutual Nearest Neighbors (MNN) (Haghverdi et al., 2018), Batch balanced kNN (BBKNN) (Polański et al., 2020) and Harmony (Korsunsky et al., 2019) have been proved to overcome the same issue and smooth out unwanted and misleading differences.

##### 2.2.1.4 Imputation

The information stored in a single cell dataset has a very sparse nature. In mathematical terms, it translates into a matrix full of zeros. Many normalization approaches do not remove them, assuming that they represent missing values to account in calculations. However, reducing their number could reduce the noise, improving the estimation of gene-gene correlations (van Dijk et al., 2018).

Currently, many tools are available to achieve this task, and the best performing ones are mainly based on deep learning algorithms (Bao et al., 2022). In this category fall DeepImpute (Arisdakessian et al., 2019) and Deep Count Autoencoder network (DCA) (Eraslan et al., 2019). The first one uses highly correlated genes of the target genes to impute the missing values, while the second can capture the nonlinear gene-gene correlation. Their application proved to improve the performance in cell clustering, DE analysis and trajectory inference.

However, when applying expression recovery, one should take into consideration that no method is perfect. Thus, any method may over- or under-correct noise in the data. Indeed, false correlation signals have been reported as a result of expression recovery (Andrews and Hemberg, 2018).

In light of this, it is hard to assess if imputation will succeed in a particular application. A reasonable approach would be to impute for visualization and avoid it to generate hypothesis during exploratory data analysis.

##### 2.2.1.5 Feature selection and dimensionality reduction

After proceeding with the “data cleaning” steps, a human scRNA-seq dataset can still contain up to 15,000 genes. Such a big and multidimensional object is, however, hard to manage and visualize. For these reasons, it is subjected to dimensionality reduction.

To go through this process it is important to keep in mind that many residual genes do not represent the data variability, which is a key feature to explore the heterogeneity of the sample, and so that we can consider them uninformative and ignorable. This process is called feature selection. A common way to reach this result is to look for highly variable genes (HVGs) by binning them by their mean expression and preserving the ones with the highest mean-to-variance ratio in each bin (Brennecke et al., 2013). Methods such as Scanpy and Cell Ranger implement functions to define the HVGs starting from log-transformed data, while others like Seurat work on the raw counts. Typically, between 1,000 and 5,000 HVGs are selected to proceed with robust downstream analysis (Klein et al., 2015).

Their identification is crucial also to proceed with the following dimensionality reduction. Indeed, common methods like the Principal Component Analysis (PCA) (Pearson, 1901; Pedregosa et al., 2011) benefit from using HVGs to define the reduced components used to summarize the dataset features in a low-dimensional space. This is possible through a linear approach which transforms a set of correlated variables into a smaller number of uncorrelated variables, called principal components (PCs), preserving as much of the data’s variation as possible. To determine the N most informative PCs, “elbow” heuristics or the permutation-test-based jackstraw method can be used (Chung and Storey, 2015; Macosko et al., 2015).

The PCA is a technique that comes from the field of linear algebra and can be used as a data preparation technique to create a projection of a dataset prior to fitting a model. Indeed, for complex datasets whose structure could not be captured by two or three PCs, non-linear combination methods such as t-distributed stochastic neighbour embedding (t-SNE) (Van der Maaten and Hinton, 2008) and Uniform Approximation and Projection (UMAP) (McInnes et al., 2018) perform better, taking advantage of PCA data.

##### 2.2.1.6 Visualization and clustering

Non-linear methods are commonly used to create a two-dimensional plot summarizing an scRNA-seq dataset from a larger number of significant components. t-SNE and UMAP are two typical solutions to achieve this task and are implemented in almost all scRNA-seq data processing toolbox. t-SNE takes a high dimensional data set and reduces it to a low dimensional graph focusing on capturing local similarity at the expense of global structure. UMAP, instead, tends to favour fully connected representations of the dataset using a cell-cell nearest-neighbour network to then estimates a low dimensional embedding of the data. The latter is largely replacing the former, although different representations could give different insights. In this perspective, it is good to know that also diffusion maps and partition-based methods exists to visualize complex data in different manners and for different applications, e.g., diffusion maps are good to make inferences in trajectory analyses, while partition-based methods approximate the topology of the data using clusters to produce a simplified “coarse-grain” visualization of the data, useful with very large datasets.

The clustering is commonly performed with the Louvain (Blondel et al., 2008) and the Leiden (Traag et al., 2019) algorithms.

The aim of this step is to define groups of cells with similar expression profiles, because these groups could represent cell types, intermediate cell states or other interesting aspects of the data.

Both methods are based on K-Nearest Neighbour approach (KNN graph) where cells are represented as nodes in a graph, each connected to its K most similar cells, obtained using Euclidean distances on the PC-reduced expression space, so that densely sampled regions of expression space will be represented as densely connected regions in the plot (Zappia and Oshlack, 2018).

Clustering can also be performed at multiple resolutions to inspect data at different levels of detail (i.e., more clusters of smaller dimensions). Moreover, the resulting groups can be iteratively subclustered to allow the identification of cell states captured within the same cluster.

##### 2.2.1.7 Cluster annotation

Once clusters have been defined, it is time to identify the represented cell populations. This can be done by defining their gene signatures through the identification of marker genes. To this end, DE testings are usually applied between two groups representing the cluster and the rest of the dataset. Next, simple statistical tests such as the Wilcoxon rank-sum test or the *t*-test are used to rank the derived genes by their difference in expression. The top-ranked genes from the respective test statistic are regarded as marker genes.

Clusters can be also annotated by comparing marker genes from the dataset with those from reference datasets *via* enrichment tests, the Jaccard index or other overlap statistics. Indeed, reference databases such as the mouse brain atlas (Zeisel et al., 2018) or the Human Cell Atlas (HCA) (Regev et al., 2017) are increasingly becoming available, facilitating cell identity annotation. Also automated methods like single cell NET (Tan and Cahan, 2019) are available to accomplish this step and speedup the annotation process, although a manual revision is always suggested due to the plasticity of cell states which sometimes could be confused with others.

##### 2.2.1.8 Trajectory analysis and metastable states

Cell clustering sometimes is not the appropriate strategy to study a dataset. Many biological processes, characterizing a dataset, cannot be described through discrete classification but rather in a more continuous way (Tanay and Regev, 2017). To achieve this result we need to apply gene dynamic models capable of ordering cells along an axis defining the time process, also known as pseudotime. This type of approach is commonly used to study processes such as development and differentiation, and it is called Trajectory analysis.

Several methods are currently available to infer trajectories of increasing complexity, from simple linear or bifurcating paths to complex graphs, trees, or more intricated trajectories.

Usually, these algorithms take the reduced or corrected data as input in order to minimize technical variation and capture only the biological one, taking advantage also of HVGs, which are used to define the consecutive states derived from transcriptional distances from a root cell. None of the available methods has been shown to overperform the others for all kinds of trajectories, although different approaches benefit different ends, as shown in previous comparative studies (Saelens et al., 2019).

For instance, the tool Slingshot (Street et al., 2018) proved to perform better when inferring linear or multifurcating trajectories, contrary to the current state-of-the-art, Monocle2 (Trapnell et al., 2014), which gives best results in more complex and branched situations, along with its later version Monocle3 (Cao et al., 2019) and the Diffusion Pseudotimes (DPT) implemented in Scanpy (Haghverdi et al., 2016).

The aforementioned python toolbox offers also the chance to reconcile the information derived from clustering and trajectory inference, by means of the Partition-based graph abstraction (PAGA) algorithm (Wolf et al., 2019). In detail, using a statistical model for cell cluster interactions, PAGA places an edge between cluster nodes whose cells are more similar than expected, generating a map representing the static and dynamic nature of the data.

As trajectory inference deals with the way the cells in our sample change according to a pseudotime, it becomes possible to define the “preferential” transcriptomic states of the process evaluating the region density. Dense regions of cells represent the so called “metastable states” which can be visualized through histograms.

Unfortunately, few of the aforementioned methods include an evaluation of uncertainty in their model, so the predicted results should be confirmed with alternative approaches to avoid method bias (Griffiths et al., 2018). A common way to achieve this goal is to infer time dynamics by measuring relative abundances of exonic and intronic reads, representing spliced and unspliced transcripts. The change of their abundance, termed RNA velocity, allows to infer the direction in which each cell is moving in expression space along with an estimate of the rate of change, unlocking new ways to study cellular dynamics by granting access to not only the descriptive state of a cell, but also to its direction and speed of movement.

Currently, two modeling approaches exist, the originally proposed “steady-state” model adopted by velocyto (La Manno et al., 2018) and the subsequently extended dynamical model implemented in scVelo (Bergen et al., 2020). The former estimates velocities as the deviation of the observed ratio of unspliced to spliced mRNA from an inferred steady-state ratio, by leading sometimes to predicition errors if the central assumptions of a common splicing rate and the observation of the full splicing dynamics with steady-state mRNA levels are violated. The latter overcomes these limitations by generalizing velocity estimation to transient systems through the application of a likelihood-based dynamical model which solves the full transcriptional dynamics of splicing kinetics.

##### 2.2.1.9 Gene expression analysis

Once the nature of each cluster is assessed, focusing on gene expression can give us a much broader idea on processes and mechanisms that differ among them. In this perspective, tools such as DE analysis and gene set enrichment analysis (GSEA) can help us investigate the molecular variability deriving from different experimental (medical treatment) or biological (different cell lines) conditions.

DE methods originate with bulk sequencing data analysis, where a few samples were compared to understand the molecular consequences of different experimental conditions. In single cell settings, the variables at stake increase as the number of cells under examination increases, due to cell-to-cell variability and biases such as dropout (Vallejos et al., 2017; Hicks et al., 2018). Tailored tools like MAST (Finak et al., 2015) or scCODE (Zou et al., 2022) are available to handle these features and perform DE on large single cell datasets in reasonable times, however, bulk DE tools, like DESeq2 (Love et al., 2014) and EdgeR (Robinson et al., 2010), have been proved to outperform some single cell counterparts if properly calibrated, but taking long times (Van den Berge et al., 2018). Uncorrected data are preferred for these applications, so it is crucial to account for confounding factors to perform a robust estimation of differentially expressed genes.

The testing result consists in a long list of genes differentially expressed between two or more conditions, sometimes hard to interpret in a meaningful way. To overcome this limitation, we can analyze them by grouping into sets based on shared characteristics, e.g., biological process and matabolic pathway. This approach, called GSEA, tests whether these characteristics are overrepresented in the candidate gene list and relies on the usage of curated databases such as the Gene Ontology (Ashburner et al., 2000; The Gene Ontology Consortium, 2017), KEGG (Kanehisa et al., 2017), String (https://string-db.org) and Reactome (Gillespie et al., 2022). Tools like gseapy (https://gseapy.readthedocs.io/en/latest/) and biomaRt (Durinck et al., 2009) are available to accomplish this task through multiple tests, querying the mentioned databases. Furthermore, novel algorithms (Vento-Tormo et al., 2018) allowed to proceed with paired ligand-receptor analyses which inspect the interaction between cell clusters.

### 2.3 Experimental design considerations

scRNA-seq has opened new avenues for the characterization of heterogeneity in a large variety of cellular systems, allowing to obtain transcriptome-wide data from individual cells. Although gene-expression profiling at single cell level has revealed an unprecedented variety of cell types and subpopulations that were invisible with traditional experimental techniques, it introduced new challenges due to the intrinsic nature of the data.

Indeed, scRNA-seq datasets show increased variability, complex expression distributions and an abundance of zeros compared to those produced in “bulk” experiments, making challenging to create broadly applicable experimental designs. In light of this, each experiment requires the user to make informed decisions before to proceed with a pondered design, which have to satisfy three principles formalized by R. A. Fisher in 1935: replication, randomization and blocking (Box, 1980).

To prepare an experiment, respecting such principles, it is good to start with a balanced block design in which samples collected from multiple conditions are evenly distributed across plates and lanes of the sequencer in order to reduce technical variation and not confound it with the biological one (Baran-Gale et al., 2018). On the opposite, processing samples separately, isolating cells from each sample onto separate plates (one for sample) and sequencing them on separate lanes (one for sample), produces a confounded design affected by additional sources of technical variation associated with batch preparation of libraries or sequencing. In this context, balanced design allow to bypass the batch correction step in the computational analysis, reducing computational times and user intervention on data.

Experimental design considerations will also be affected by the various protocols and platforms available for scRNA-seq. For instance, full-length capture or 3′ methods offer different way to explore sample characteristics.

As example, in an observational study setting, working with high numbers of cells could be the best solution to get insights on the transcriptional heterogeneity of the sample. To this aim, 3′ methods represent the best solution allowing to capture higher amounts of cells (100–1,00,000) and quantify their transcriptomes in a more simple and precise way, thanks to the usage of UMIs. On the other hand, to conduct more “in depth” observations or study genetic alterations (SNPs, structural variants) in the transcribed fraction, full-length approaches are more well suited, benefiting from a higher capture efficiency and a more precise information, but at cost of a minor number of cells (96–384). Therefore, more reads will be required for more refined tasks (Pollen et al., 2014; Wu et al., 2014), such as fully characterizing transcript structure, estimating the expression of rare isoforms, or distinguishing cells on the basis of subtle differences, while fewer reads but larger cell numbers may be preferred when mapping out a large population, searching for rare but distinct cell types, or pooling cells *in silico* to obtain average gene-expression clusters. According to this, if we design an experiment to search for a rare cell population, we have to take into account the number of cells that need to be sequenced to get such a population. This parameter can be estimated based on the expected heterogeneity of all cells in a sample, the minimum frequency expected of the rare cell type within the sample and the minimum number of cells of each type desired in the resulting data set.

In case no prior knowledge about the heterogeneity of the cell population is available, a practical solution is to perform the study with a high cell number and lower sequencing depth, and then perform pre-purification of the interested cells by fluorescence-activated cell sorting (FACS) with in-depth sequencing.

Another relevant difference between the two protocols relates to the UMIs usage. Indeed, full-length approaches make the inclusion of UMIs difficult, as each full-length transcript is fragmented following reverse transcription, and each fragment would need to be linked to the single UMI for that transcript. On the other hand, 3′ methods, like the 10x Genomics system, include a 10/12 bp UMI in each read at the beginning of the protocol, facilitating the molecule counting and the evaluation of sequencing saturation through the analysis of UMI duplicates. Moreover, the use of UMI has an impact on normalization procedure, since they are a consistent means to correct for amplification bias.Overall, several factors need to be considered before choosing a method for scRNA-seq. Whatever the design, it is always beneficial to record and retain information on as many factors as possible to facilitate downstream diagnostics.

## 3 Biomedical applications

Modern cellular and molecular biology knowledge is largely derived from RNA-seq experiments which allowed to understand the complexity of the dynamics responsible for metabolic alterations, fueling much discovery and innovation in the field of medicine over recent years.

The evolution of such techniques was driven by the development of protocols and devices capable of extracting transcriptomic information from an ever increasing number of single cells, laying the groundwork for heterogeneity-oriented studies.

The chance to dissect a sample in its composing cell lines opened up new perspectives in clinical studies oriented to the discovery of rare cell populations involved in the onset and evolution of diseases such as tumors. A proof of this assertion comes from Ramsköld et al., in 2012 and Patel et al., in 2014, which studied, for the first time (Ramsköld et al., 2012; Patel et al., 2014), the compositional architecture of melanoma and glioblastoma samples at single cell level. In the wake of them, an increasing number of studies and researchers have started exploiting the technique to successfully characterize cell populations in a variety of tumors (Dago et al., 2014; Ting et al., 2014; Puram et al., 2017; Zhao et al., 2020; Pal et al., 2021; Tian et al., 2022), defining their role into the disease process and their identity through the assignment of gene signatures (Young et al., 2018; Peired et al., 2020). Other contributions to the field comes from the integration of scRNA-seq and Copy Number Variant (CNV) detection. Tirosh et al., in 2016, successfully applied this technique to get new insights on intra- and interindividual, spatial, functional and genomic heterogeneity in melanoma cells, as well as details related to the tumor microenvironment and the cells populating it, validating the presence of a dormant drug-resistant population (Tirosh et al., 2016).

Similarly, in 2018, Fan et al. took advantage of CNVs and Loss of Heterozygosis (LOH) to identify and characterize the transcriptional programs which drive the distinct genetic subclones in a tumor sample (Fan et al., 2018).

Also in the neurological field, the scRNA-seq succeeded, revealing the heterogenous nature of brain cells involved in Alzheimer’s disease and the different outcomes related to their different gene expression patterns (Mathys et al., 2019). In this contest, Lodato et al. exploited single cell sequencing to identify Single Nucleotide Variants (SNVs) in neuronal cells, demonstrating how somatic mutations can be used to reconstruct the developmental lineage of neurons, which live for decades in a postmitotic state accumulating mutations responsible for the creation of nested lineage trees and the relative polyclonal architecture (Lodato et al., 2015).

While, for blood, liver and heart samples, the introduction of trajectory analyses have provided new insights on differentiation processes, allowing to trace the fate of progenitor cells revealing the plasticity of their transcriptome through the identification of new transitional cell states (Jia et al., 2018; Popescu et al., 2019; Liang et al., 2022). However, the regulatory networks driving these processes are more complex and characterized by confounding factors like redundancy and nonlinear cross talk between pathways, e.g., developmental and signaling factors in the immune system. An unbiased approach to elucidate such a circuits and their alterations are the perturbation studies, which, by making use of the massive parallelism of single cell technologies merged with CRISPR-mediated editing, allow to knockout multiple target genes simultaneously producing different cell responses useful to clarify the function of multiple factors and their interactions in tens of thousands of cells (Adamson et al., 2016; Dixit et al., 2016; Jaitin et al., 2016). To extend this application to the analysis of multiple unrelated individuals, new methods that harness natural genetic variation were developed. Tools like demuxlet (Kang et al., 2018) determine the sample identity of each droplet, using genotyping data (SNPs), to characterize inter-individual variation and cell-type-specific genetic control of gene expression. Similarly, Van der Wijst et al. used SNP data to characterize alterations of gene co-expression pathways, focusing also on celltype-specific expression quantitative trait loci (eQTLs) (van der Wijst et al., 2018), promoting a new way to identify genetic variants that impact regulatory networks.

Another hot topic is damage recovery, since a better understanding of these mechanisms could allow us to identify the players involved in success or fail of such processes, offering new hints in the development of better diagnostic tools, prognostic biomarkers and signaling pathways amenable to therapeutic targeting (Kirita et al., 2019; Melica et al., 2022).

## 4 Future perspectives and conclusion

Single cell RNA sequencing was proven to be a cutting-edge technology in life sciences over the past decade. This field is developing remarkably rapidly and numerous easily accessible commercial solutions capable of characterizing hundreds of thousands of cells in parallel in reasonable times at competitive costs are currently available, making scRNA-seq much better suited for biomedical research and for clinical applications.

The spread of these devices fueled much discovery and innovation also in the computational biology field, promoting the development of novel approaches to extract information from the data produced by such technologies, and algorithms capable of analyzing them, scaling computational times more favorably with the dataset size. Moreover, along with RNA profiling, single cell technologies are currently employed to acquire information about multiple types of molecules in parallel, promoting the so-called “multimodal profiling”. In fact, today it is possible to integrate information related to chromatin accessibility (Cusanovich et al., 2015), methylation state (Angermueller et al., 2016), cell-surface proteins (Stoeckius et al., 2017), to reveal the full-scale complexity of biological systems. Also, the developmental trajectories can be studied in a more precise way by matching the single cell technologies with CRISPR-Cas9 based genome editing. Methods such as scGESTALT (Raj et al., 2018) and LINNAEUS (Spanjaard et al., 2018) allow to simultaneously characterize molecular identities and lineage histories of thousands of cells during development and disease through the analysis of lineage barcodes, generated by genome editing.

However, high-throughput techniques come with the expense of decreased molecule capture rates, and future methods need to better balance cell numbers with cell resolution. Furthermore, with the future development of new and better bioinformatic tools, the individual tool recommendations presented here will require updates, yet the general considerations regarding the stages of data processing should remain the same.

Spatial dimension of single cell transcriptomics also represents an exciting field because, although novel and more precise technologies are becoming available (Eng et al., 2019), it presents several common challenges that limit its applications, including non-single cell resolution, relatively low sensitivity, high cost and labor-intensive process.

In conclusion, we have presented a brief and concise overview of single cell RNA sequencing technology and its applications. The continuous development of the technology will broaden its adoption in clinical and personalized medicine.
